# Hysterosalpingographic evaluation of human immunodeficiency virus-infected and uninfected infertile women

**DOI:** 10.4102/sajr.v24i1.1767

**Published:** 2020-03-24

**Authors:** Dolongo C. Onyangunga, Jagidesa Moodley

**Affiliations:** 1 Department of Radiology, School of Clinical Medicine, University of KwaZulu-Natal, Durban, South Africa; 2 Women’s Health and HIV Research Unit, Department of Obstetrics and Gynaecology, School of Clinical Medicine, University of KwaZulu-Natal, Durban, South Africa

**Keywords:** hysterosalpingography, infertility, HIV, sexually transmitted diseases, fallopian tubes, radiology

## Abstract

**Background:**

Hysterosalpingography (HSG) is an outpatient fluoroscopy-guided procedure that evaluates the uterine cavity and fallopian tube patency in infertile women. Its cost-effective use is being challenged with the human immunodeficiency virus (HIV) burden in KwaZulu-Natal, which characteristically affects multiple organs.

**Objectives:**

The aim of this study was to describe the HSG findings in a group of HIV-infected and uninfected infertile women.

**Method:**

This was a retrospective study conducted over a 4-year period (2012–2016) in which the HSG images and reports of 178 infertile patients from records of the Radiology Department were re-reviewed for abnormalities of the cervix, uterus and fallopian tubes. Their clinical data and radiological findings were entered into a pre-coded data sheet and analysed.

**Results:**

The frequency of HIV infection amongst patients with infertility was found to be 32.6%. Forty-four patients were on antiretroviral therapy at the time of the HSG examination, whereas three had not yet started treatment. From the 178 HSG reports, 109 (61.2%) were abnormal. Tubal pathologies were the most common abnormalities, accounting for 79 of the 109 cases and was higher in HIV-infected women than in HIV-uninfected women (*p* = 0.001). Uterine filling defects were demonstrated in 13 of the 109 cases. There were two cases of cervical abnormalities.

**Conclusion:**

The study demonstrated that tubal abnormalities were the most common findings amongst infertile women undergoing HSG and occurred predominantly in HIV-infected patients.

## Introduction

Infertility is defined as the incapacity of a couple at reproductive age to conceive after regular and unprotected sexual intercourse within 2 years.^1^ It may be classified as primary infertility for a couple who never conceived or secondary infertility when occurring after a previous pregnancy.

Infertility remains a serious health concern with sociocultural implications, particularly in women of African ancestry.^2^ In Africa, 30% – 40% of couples present with infertility.^3^ The major cause of infertility in South Africa (SA) is sexually transmitted disease, which leads to genital tract pathology including endometritis and salpingitis, complicated by tubal mucosal damage and blockage. The human immunodeficiency virus (HIV) epidemic in SA over the last three to four decades has compounded the impact of sexually transmitted infections on infertility.^4,5,6^ However, treatment of HIV infection has been reported to reduce infertility rates.^7,8^

A variety of imaging techniques including hysteroscopy, ultrasonography (US), hysterosalpingography (HSG) and pelvic magnetic resonance imaging (MRI) are available to evaluate the patency of the female genital tract for the investigation of infertility. The unavailability of expertise and the costs of imaging techniques, such as MRI and diagnostic laparoscopy, make HSG the most commonly used first-line imaging technique for evaluating female infertility in district and most regional hospitals in SA.

To the best of our knowledge, there are no local reports on HSG findings in HIV-infected women with infertility. This is of importance for the KwaZulu-Natal province, which carries the highest burden of the HIV pandemic, with rates of approximately 37% in antenatal attendees.^9^ Therefore, the aim of this study was to report on HSG findings in a setting of high HIV infection rates.

## Methods

### Study design

This was a descriptive, retrospective study covering a 4-year period, from 01 January 2012 to 31 December 2016, in the Radiology Department of a Central Hospital in Durban, KwaZulu-Natal. The radiological information system of the hospital was used to obtain the relevant clinical records and imaging findings of patients referred for HSG as part of an infertility work-up during the study period. Each included patient had prior US of the pelvis and laboratory tests for ovulation. Hysterosalpingography was performed according to standard protocols, with the images reviewed by both a gynaecologist and a radiologist.^10^ The final reports were documented by the radiologist. The report and final images were archived in a Radiology Information Database. For the purpose of this study, both the reports and the images were re-reviewed by the author, a registrar in radiology, and by a specialist radiologist with 4 years of experience.

The socio-demographic and clinical information was recorded on a structured data sheet. The documentation of the HIV status was considered absolute criteria for inclusion. We considered the risk factor profile of women with secondary infertility as well.^11^ Women with incomplete clinical information, other proven causes of infertility and a history of tuberculosis or records of anti-tuberculosis treatment were excluded from the study.

All statistical analysis was performed using Statistical Package for the Social Sciences (SPSS version 25, IBM Corp, 2017). The results were expressed as mean and standard deviation. The *Z*-test was used for the difference between means. A *p*-value of < 0.05 was considered statistically significant.

## Results

The records of 178 infertile women with complete information who underwent HSGs were retrieved. The mean (standard deviation [s.d.]) age and duration of infertility of the patients were 33.3 ± 4.7 years and 5.64 ± 4.1 years, respectively. Amongst them, 64.6% had primary infertility and 35.4% had secondary infertility. Hysterosalpingography reports were normal in 38.8% (69/178) of patients, whilst the remaining 61.2% patients (109/178) had at least one abnormal HSG finding. Subgroup analysis showed that 120 (67.4%) women were HIV-uninfected and 58 (32.6%) were HIV-infected. Forty-four patients were on antiretroviral therapy at the time of HSG examination, whereas three had not yet started treatment. The demographic and clinical profiles of the infertile women are presented in Tables 1 and 2.

**TABLE 1:** Demographic and clinical profile of infertile women.

Variable	Mean ± s.d.	*n*	%	Minimum	Maximum
**Age groups (years)**	**33.3 ± 4.7**	**-**	**-**	**21**	**43**
< 25	-	6	3.4	-	-
25–30	-	41	23.0	-	-
31–40	-	124	69.7	-	-
> 40	-	7	3.9	-	-
**Parity groups**	**0.56 ± 0.88**	**-**	**-**	**0**	**3**
0	-	115	64.6	-	-
1	-	37	20.8	-	-
2	-	16	9.0	-	-
3	-	10	5.6	-	-
**Type of infertility**					
Primary	-	115	64.6	-	-
Secondary	-	63	35.4	-	-
Duration of infertility (years)	5.64 ± 4.1	-	-	1	18
**Previous miscarriage**					
Yes	-	12	6.7	-	-
No	-	166	93.3	-	-
**Previous surgery**					
Yes	-	41	23.0	-	-
No	-	137	77.0	-	-
**Previous pelvic inflammatory disease**					
Yes	-	43	24.2	-	-
No	-	135	75.8	-	-

s.d., standard deviation.

**TABLE 2:** Demographic and clinical profile of infertile women based on human immunodeficiency virus status.

Variable	HIV-infected			HIV-uninfected			*p*
	Mean ± s.d.	*n*	%	Mean ± s.d.	*n*	%	
**HIV status**							
CD4 (*n* = 19)	481.9 ± 258.3	58	32.6	-	120	67.4	-
**HIV treatment (*n* = 44)**							
FDC	-	24	13.5	-	-	-	-
HAART	-	20	11.2	-	-	-	-
**Type of infertility**							
Primary	-	31	-	-	84	-	-
Secondary	-	27	-	-	36	-	-
**Age groups (years)**	**33.4 ± 5.1**	**-**	**-**	**33.2 ± 3.7**	**-**	**-**	**0.7**
< 25	-	0	-	-	6	-	-
25–30	-	12	-	-	29	-	-
31–40	-	45	-	-	79	-	-
> 40	-	1	-	-	6	-	-
**Parity (groups)**	**0.5 ± 0.87**	**-**	**-**	**0.7 ± 0.9**	**-**	**-**	**0.07**
0	-	31	-	-	84	-	-
1	-	14	-	-	23	-	-
2	-	11	-	-	5	-	-
3	-	2	-	-	8	-	-
**Duration of infertility (years)**	**5.7 ± 4.2**	**-**		**5.5 ± 3.8**			**0.8**
**Miscarriage**	-	10	-	-	2	-	**0.1**
**Pelvic inflammatory disease**	-	24	-	-	19	-	**0.001**

HIV, human immunodeficiency virus; FDC, full drug combination; HAART, highly active antiretroviral therapy; *n*, number; s.d., standard deviation; CD4, cluster of differentiation4.

Of the 41 women who had previous pelvic surgery, 27 were HIV-uninfected and 14 were HIV-infected. The association with HIV infection was significant (*p* = 0.047). Forty-three (24.2%) women provided a history of pelvic inflammatory disease (PID) and 24 (55.81%) of those were HIV-infected. Pelvic inflammatory disease and HIV were also significantly associated (*p* = 0.001).

Sixty-three patients presented with secondary infertility before undergoing HSG. Previous caesarean delivery was performed in 29 of these patients presenting with uterine and/or tubal abnormalities at HSG. Human immunodeficiency virus infection was more frequent in the caesarean delivery group than in the vaginal delivery group (*p* = 0.000).

Tubal pathologies were the most common abnormalities and accounted for 79/109 (72.5%) of the abnormal findings. They presented as bilateral occlusions or unilateral tubal occlusions with hydrosalpinx. The next most common abnormality was seen in the uterine cavity, with 13 filling defects. The majority (14) of filling defects were confirmed on pelvic US as air bubbles and not considered as a pathology; 10 were submucosal fibroids and three were congenital uterine malformations. There was one uterine synechia. Cervical pathologies included elongated cervix and stenosed cervix, where the procedure was then abandoned.

Ten HIV-infected patients (17.2%) had more than one documented cause of infertility. Tubal pathologies were the only cause of infertility that was significantly associated with HIV infection (*p* = 0.001). The HSG findings are presented in Table 3.

**TABLE 3:** Hysterosalpingography findings.

Abnormalities	Total (*n* = 178)	HIV-infected (*n* = 58)		HIV-uninfected (*n* = 120)		*p*
		*n*	%	*n*	%	
**Cervix (*n* = 2)**						
Elongated cervix	1	1	1.7	0	0	-
Stenosed cervix	1	1	1.7	0	0	-
**Uterus (*n* = 28)**						
Filling defect	13	6	10.3	7	5.8	-
Uterine synechiae	1	1	1.7	0	0	-
Air bubbles	14	-	-	-	-	-
**Tubal pathology (*n* = 79)**	**79**	**46**	**79.3**	**33**	**28.3**	0.001 *
Unilateral hydrosalpinx	18	7	12.1	11	9.2	0.5
Bilateral hydrosalpinx	11	8	13.8	3	2.5	0.3
Bilateral distal occlusion	5	5	8.6	0	0	-
Unilateral distal occlusion	15	10	7.2	5	4.2	0.05
Bilateral proximal occlusion	8	4	6.9	4	3.3	0.7
Unilateral proximal occlusion	14	4	6.9	10	8.3	0.7
Bilateral proximal/distal occlusion	4	4	6.9	0	0	-
Unilateral proximal/distal occlusion	4	4	6.9	0	0	-

HIV, human immunodeficiency virus.

* , significant at *p* < 0.05.

## Discussion

The main findings of this study showed that a substantial proportion of patients who had HSGs were HIV-infected (32.6%). The study confirmed a smaller number of HIV-infected infertile women <25 years old and a larger proportion between 25 and 40 years of age. Although infertility affects all reproductive ages, it was also not documented in any HIV-uninfected patients less than 25 years of age. In a similar study conducted by Heis et al.,^12^ the overall mean age was 31.5 years (s.d. 5.9 years), with maximum occurrence at 18–46 years and a mean (s.d.) infertility duration of 4 ± 3.4 years. Panti^13^ reported a mean age of 28.9 ± 6.5 years and a mean duration of infertility of 7.47 ± 1.6 years. Chen and Walker^14^ reported that the age-specific fertility rates of HIV-infected women are reduced as compared to uninfected women, except for those in the 15–19 years age range where fertility is said to be higher based on reports from many countries.

In this study, the incidence of primary infertility was higher than that of secondary infertility, which is in accordance with other studies.^13,14,15^ However, contradictory findings where secondary infertility was more common than primary infertility have also been documented.^6,16^ We did not find any significant statistical association between the duration of infertility and HIV.

The frequency of HIV infection in the study was 32.6%. This prevalence rate is much higher than previous reported values from Gabon (9.3%),^17^ Tanzania (18.2%)^17^ and SA (20%).^18^ The high rates of HIV are probably because KwaZulu-Natal has the highest HIV burden in SA; the rate amongst antenatal attendees is 37%.^8^

It should be noted that 25% of the HIV-infected infertile women in this study had not initiated anti-retroviral (ARV) treatment. Although no side effects or adverse events of HSG were noted in the study for both infected and uninfected women, the study was not designed to evaluate the complications in HIV-infected women following HSG. This might be regarded as a limitation of the study. It is plausible that any invasive procedure of the female genital tract in HIV-infected women may cause infective complications. This needs further investigation and it seems intuitive that gynaecologists initiate ARVs prior to any investigations for infertility. We only found one study in the current literature that has evaluated HSG procedures in HIVpatients.^19^

The results of this study revealed that 38.8% of the patients had normal HSGs; however, our finding is lower than the 49.2% reported previously in a similar study in Nigeria.^8^ This may be related to the HIV burden in the study sample. Other studies have recorded 16.6% and 29.1% of normal findings.^13,20,21^

Tubal factor infertility was seen in 79.3% of our HIV-infected patients. Some HIV-infected patients (6.2%) had more than one documented cause of infertility. Tubal factor was the only cause of infertility that was significantly associated with HIV infection (*p* = 0.001). Yahya et al.^7^ reported tubo-peritoneal factor as the most common cause of infertility (seen in 81% of HIV-infected patients) and the association as statistically significant, with a *p*-value of 0.048. Similarly, Adegoke et al.^8^ reported that tubo-peritoneal abnormalities were more common amongst infertile women infected with HIV as compared to those without HIV infection. Adesiyun et al.^4^ also reported a study on HSG and found that distal tubal occlusion with hydrosalpinx was mainly associated with HIV infection.

### Limitations of the study

There was no follow-up of the patients to monitor any long-term complications related to the procedure. Furthermore, there was no record of the duration or initiation of ARV regimen. Many patients were excluded from the study because of a history of tuberculosis or anti-tuberculosis treatment, even though there was a concurrent positive medical history of HIV. It is important to note that the infertile women were investigated without examining their spouses; thus, it could be that their infertility is related to their partners. This is a major limitation of this study.

## Conclusion

This study has shown that HSG remains an important diagnostic tool in the evaluation of the infertile women, particularly in public-sector health facilities in SA, where there is a lack of equipment, technology and expertise. Hysterosalpingograms are cheap, quick and easily accessible. We demonstrated that tubal abnormalities were the most common findings amongst infertile women undergoing HSG, occurring predominantly in HIV-infected patients.
